# Parliament and the Professions

**Published:** 1921-07-23

**Authors:** 


### Parliament and the Professions.

### The Drought and National Health.

Sir Walter de Freece asked the Minister of Health if reports showed an increase of sickness due to the drought and excessive heat, and what precautionary measures against the outbreak of infectious diseases had been taken. Sir Alfred Mond stated that he had received no report on the question, but a circular is being issued to the local authorities as to the danger to young children of epidemic diarrhœa in present weather conditions.

### Steyning Infirmary, Brighton.

Mr. T. Griffiths asked the Minister of Health about the discharge of two nurses from the Poor-law institution at the Steyning Infirmary, Brighton, and Sir Alfred Mond replied that he is informed by the Board of Guardians that they appointed a special committee to inquire into the case, who came to the conclusion that the charge had been proved, and that the nurses had been guilty of great unkindness to a patient and also insubordination. The nurses would be furnished with an extract from the minutes showing that they had passed their examinations.

### State Support of Irish Hospitals.

The Chief Secretary for Ireland explained the grants made to the Dublin hospitals from the Imperial Exchequer. An agreement in a clause of the Act of Union bound the Parliament of the United Kingdom to continue certain grants made by the Irish Parliament for charitable purposes. These grants included contributions to the following hospitals in Dublin: Hospital for Incurables, Home of Industry, Westmorland, and the Meath Hospital. The Irish Parliament also contributed generously to the construction of the Rotunda Hospital. The English Government was bound to continue the grants only for twenty years, but, taking a wide view, the contract has been extended. The present grants were finally fixed by the Select Committee on Irish Estimates in 1854, and modified by the report of the Royal Commission of 1855.

